# Preparation of biotemplated Fe_3_O_4_ nanoparticles and evaluation of RF-induced heating efficiency for targeted hyperthermia

**DOI:** 10.1039/d5ra03372a

**Published:** 2025-10-06

**Authors:** Y. Ranjith Kumar, Pragya Trivedi, Avijit Jana, D. Suman, M. Vasundhara

**Affiliations:** a Polymers and Functional Materials Department, CSIR-Indian Institute of Chemical Technology Hyderabad-500007 India mvas@iict.res.in +91 9496445333; b Department of Natural Products and Medicinal Chemistry, CSIR-Indian Institute of Chemical Technology Hyderabad-500007 India; c Academy of Scientific and Innovative Research (AcSIR) Ghaziabad-201002 India; d Department of Biomedical Engineering, Osmania University Hyderabad-500007 Telangana India

## Abstract

In this study, we report the synthesis of Fe_3_O_4_ nanoparticles (NPs) employing a mild sol–gel synthesis method with Fe-nitrate precursors and egg deutoplasm fluid, a bio-template, as stabilizing agent. The Fe_3_O_4_ NPs were synthesized with varying concentrations of the stabilizing agent to determine the optimal conditions. The synthesized Fe_3_O_4_ NPs were comprehensively characterized to evaluate their structural, morphological, optical, and magnetic properties. The characterization techniques used were X-ray diffraction (XRD), Raman spectroscopy and X-ray photoelectron spectroscopy (XPS), Transmission electron microscopy (TEM), Fourier transform infrared (FTIR) spectroscopy, UV-Visible spectroscopy and Vibrating Sample Magnetometry (VSM). Magnetic measurements were also conducted at room temperature to understand the magnetic behaviour, a crucial property for biomedical applications such as hyperthermia and targeted drug delivery. To explore their potential for hyperthermia applications, the Fe_3_O_4_ NPs were exposed to radio-frequency (RF) for evaluating their heating efficiency. The Fe_3_O_4_ NPs exhibited significant RF absorption, leading to effective thermal conversion and achieving the target hyperthermic temperature of 42 °C, which is essential for cancer treatment. The ability of the synthesized Fe_3_O_4_ NPs to generate localized heat in response to RF energy underscores their potential for precise and controlled hyperthermic therapy. This study highlights the importance of optimizing synthesis conditions to tailor the magnetic properties and heating ability of Fe_3_O_4_ NPs for biomedical applications. The findings demonstrate that bio-templated Fe_3_O_4_ NPs offer a promising approach for targeted cancer therapy by leveraging RF-induced heating for localized and effective treatment.

## Abstract 2

Successfully synthesized Fe_3_O_4_ nanoparticles using a bio-templating approach. Achieved controlled nanoparticle size and morphology. Investigated the effect of bio-template concentration on magnetic properties and heating performance.

## Graphical abstract

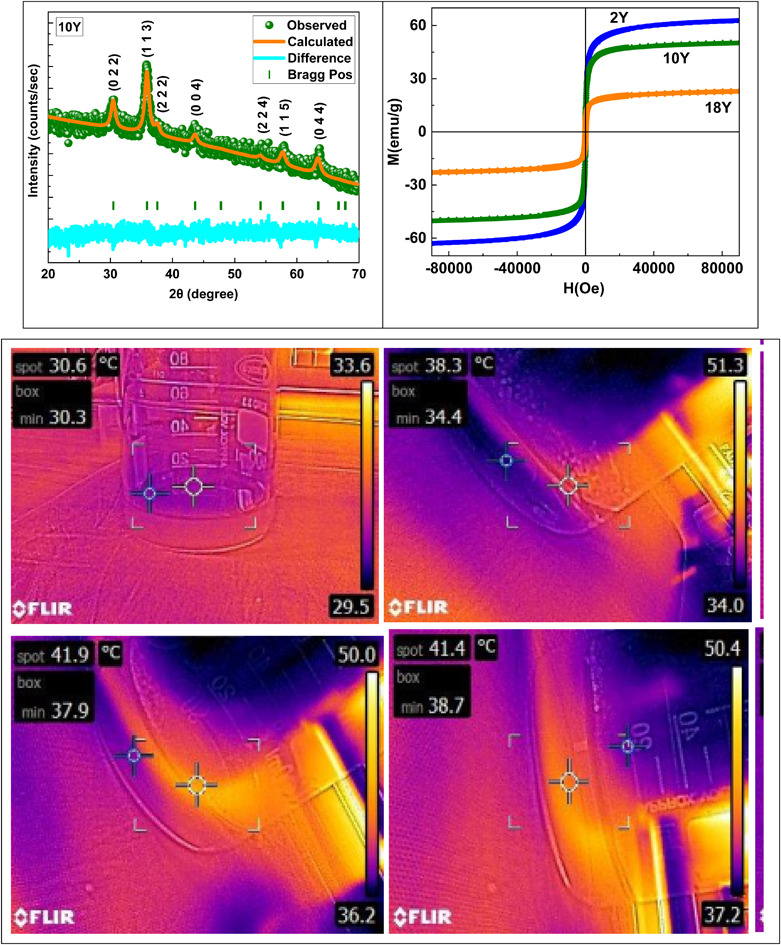

## 1. Introduction

Iron oxide nanoparticles (NPs) have shown exceptional promise in biomedicine, particularly for cancer treatment through hyperthermia, enhanced imaging in magnetic resonance imaging (MRI), and remotely controlled targeted drug delivery. They are especially valued for their outstanding biocompatibility, making them a focal point of research aimed at advancing medical technologies and achieving commercial success.^1–6^

Magnetic hyperthermia, in which magnetic nanoparticles generate heat under an alternating magnetic field (AMF), is one of the most promising therapeutic approaches for cancer treatment, as localized heating of tissues can enhance the effectiveness of chemotherapy and radiotherapy.^7,8^ However, translating this approach to clinical use faces several important challenges, including ensuring biocompatibility to avoid adverse biological effects, achieving efficient heat transfer to deep or irregularly shaped tumours, selecting the most suitable delivery route (systemic *vs.* local injection) to ensure targeted accumulation, determining the optimal nanoparticle dosage for a balance between therapeutic efficacy and safety, and maintaining controlled biodistribution to minimize off-target heating.^9–11^ The intrinsic physicochemical properties of the nanoparticles – such as mineralogy, crystallinity, particle size and size distribution, aggregation behaviour, surface area, and surface coating play a pivotal role in determining heating efficiency, biodistribution, stability, and safety in hyperthermia applications.^12^ Fine-tuning these parameters is therefore critical for enhancing performance and minimizing side effects.

Hyperthermia or pasteurisation using magnetic particles has a long history; the concept was mentioned in a paper by Goldenberg and Tranter in 1952 and tested on tumours in dogs by Gilchrist in 1957.^13,14^ Renewed interest appeared from the 1980s with numerous groups investigating smaller particles coated for increased biocompatibility. Shortwave diathermy (SWD) is one of the widely used medical technologies that involve the use of high-frequency electromagnetic waves to produce deep tissue heating. Iron oxide NPs with SWD provide a novel strategy for targeted hyperthermia therapy.^15–17^ Several alternative therapeutic strategies have been reported, including microwave hyperthermia by Zhang *et al.* (2025),^18^ photothermal therapy by Duan *et al.* (2023),^10^ ultrasound hyperthermia by Zhu *et al.* (2023),^11^ and radiofrequency ablation by Rejinold *et al.* (2015).^12^ Compared to other hyperthermia strategies, magnetic nanoparticle-based heating provides non-invasive, depth-controllable, and targeted thermal therapy. Many preclinical studies have shown that positive temperature differences can be induced between tumours and normal tissue. This implies a more localised mechanism of action than whole-tumour hyperthermia in these cases. Iron oxides commonly refer to three primary forms: magnetite (Fe_3_O_4_), hematite (α-Fe_2_O_3_), and maghemite (γ-Fe_2_O_3_). Among these, Fe_3_O_4_ has gained prominence due to its superior magnetic properties and ease of formation at lower temperatures. Fe_3_O_4_, a naturally occurring mineral with a face-centered cubic (FCC) structure, features a mixed-valence state of Fe^2+^ and Fe^3+^, confirming to the inverse spinel group with the formula [Fe^3+^]_tetra_[Fe^2+^ Fe^3+^]_octa_O^4^.^19,20^ Its unique structure and magnetic behavior have led to its use in drug delivery systems, cell separation, MRI enhancements, and various therapeutic applications.^21–23^

The synthesis of Fe_3_O_4_ NPs has been achieved through methods such as co-precipitation, micro emulsion, thermal decomposition, hydrothermal treatment, ultrasonic methods, and sol–gel processes.^24,25^ Among these, the sol–gel technique stands out for its ability to produce not only iron oxide NPs, but also diverse metal oxide nanostructures because of several advantages, such as cost-effectiveness, ease of use, homogeneity, excellent phase control and capability to produce precise stoichiometric control at relatively low temperatures.^2,22,26,27^ Recent advancements in nanomaterials synthesis have focused on incorporating natural extracts as templates for controlled morphology and size. Plant-based extracts from sources such as tea, Aloe vera, apples, and peppers have proven effective for the formation of nanoparticles.^28–30^ Compared to conventional chemical methods, biomolecule-mediated synthesis offers several benefits, including reduced energy consumption, and the use of non-toxic solvents. These biomolecules also play a critical role in stabilizing nanoparticles, preventing aggregation, and influencing their physical and chemical properties by moderating the reduction kinetics of metal precursors.^31–34^ Egg yolk, or deutoplasm, surrounded by egg white (albumen) in eggs, contains approximately 40 types of proteins that provide health benefits and exhibit functional properties like emulsification, foaming, gelling, and binding adhesion. These proteins, which are water-soluble, have a natural affinity for metal ions, including manganese (Mn), iron (Fe), copper (Cu), zinc (Zn), and nickel (Ni).^28–31^ The interaction of these metal ions with egg yolk has been explored to create novel nanomaterials with distinctive properties.^35,36^

The novelty of this study lies in systematically exploring how varying yolk concentrations on Fe_3_O_4_ NPs influence their thermal response under different saline conditions. While several studies have reported on magnetic nanoparticle hyperthermia, the combined effect of surface coating thickness (biological yolk concentration) and medium salinity on heating efficiency has received little attention. This approach provides new insights into how nanoparticle coatings not only stabilize particles but also modulate ionic interactions in different environments, directly affecting both magnetic and heating performance. Such understanding is crucial for hyperthermia applications, as the *in vivo* environment is highly heterogeneous in ionic strength and composition. By addressing this gap, our study bridges the difference between idealized laboratory conditions and realistic physiological scenarios, thereby enhancing the translational potential of magnetic nanoparticle-based hyperthermia.

In this study, Fe_3_O_4_ NPs were prepared using the sol–gel method, employing chicken egg yolk (deutoplasm) as both reducing agent the stabilizing medium and ferric nitrate [Fe(NO_3_)_3_·9H_2_O] as precursor. Furthermore, a comprehensive set of characterizations was performed to thoroughly assess the synthesized NPs and hyperthermia performance tests (SAR measurements and infrared thermal imaging) to evaluate their heating efficiency under an alternating magnetic field (AMF).

## 2. Experimental section

### 2.1. Materials

The ferric nitrate nonahydrate [Fe(NO_3_)_3_·9H_2_O] (analytical reagent grade, 98% purified) used is supplied by Sisco Research Laboratories (SRL) Pvt. Ltd, India and used without any further purification. The chemicals also used are hydrochloric acid (HCl 37%) (in UV characterization), distilled water (D.I, water). Fresh chicken eggs were brought from local poultry farm.

### 2.2. Preparation of Fe_3_O_4_ NPs

Fe_3_O_4_ NPs are synthesized in deutoplasm solution with Fe(NO_3_)_3_·9H_2_O salt as precursor by engaging simple sol–gel synthesis technique. The medium, solution is prepared by adding 2 ml of deutoplasm in 98 ml of distilled H_2_O and mixed well to make it a homogeneous mixture. The 15.756 g of Fe(NO_3_)_3_·9H_2_O is added to the above homogeneous deutoplasm solution and allowed for stirring on hot plate magnetic stirrer with slow evaporation at 70 °C until a residue of brown gel is appeared. The gel residual is dried at 100 °C, finely grinded and calcined at 400 °C for 4 h with a ramp rate of 3 °C min^−1^. The obtained sample is denoted as 2Y (2 ml of Yolk or Deutoplasm in 98 ml of distilled H_2_O). Similarly, the Fe_3_O_4_ NPs were synthesized with different concentrations of deutoplasm solution denoting the samples as 6Y (6 ml of yolk in 94 ml of distilled H_2_O), 10Y (10 ml of yolk in 90 ml of distilled H_2_O), 14Y (14 ml of yolk in 86 ml of distilled H_2_O) and 18Y (18 ml of yolk in 82 ml of distilled H_2_O).

### 2.3. Material characterization

The structural, chemical compositions and chemical states of the samples were determined through X-ray diffraction (XRD), Raman Spectroscopy (RS) and X-ray photoelectron spectroscopy (XPS) analyses. XRD measurements were carried out using a PANalytical-Empyrean diffractometer equipped with Cu Kα radiation at *λ* = 1.5404 Å with step size of 0.020°, covering scan angle range of 10–80°. Rietveld refinement of the measured diffraction data was performed using FullProf software. The Raman scattering study was performed using a T64000 triple monochromator (Horiba Jobin Yvon system) having a 514 nm laser in backscattering geometry. XPS analysis was performed using a Kratos AXIS Supra instrument. The crystal structures were further observed and lattice spacing is calculated using high-resolution transmission electron microscopy (HRTEM, FEI Talos F200X TEM/STEM). The optical properties like energy band gap and absorbance of infrared light of the samples are examined using Shimadzu UV-2401 UV-visible spectroscopy (Shimadzu Scientific Instruments, Kyoto, Japan) and Fourier transform infrared spectral data is recorded in the range of 400–4000 cm^−1^ using PerkinElmer spectrum 100 FT-IR Spectrometer. Magnetic studies at 300 K were measured utilizing Vibrating sample magnetometer attached to the Physical Property Measurement System from Quantum Design, PPMS Dynacool model.

### 2.4. Cytotoxicity assessment (MTT assay)

The *in vitro* cytotoxic profile of the formulated nanoparticle was assessed on the normal human embryonic kidney (HEK293) cell line using the MTT assay (3-(4,5-dimethylthiazol-2-yl)-2,5-diphenyltetrazolium bromide). HEK293 cells were cultured in Dulbecco's Modified Eagle's Medium (DMEM) supplemented with 10% fetal bovine serum (FBS) and seeded into 96 well plates at a density of 5000 cells per well. After allowing 24 h for cell adherence, the cells were exposed to a concentration gradient of the nanoparticle (0, 10, 20, 40, 60, 80, and 100 μg ml^−1^) and incubated for 24, 48, and 72 h at 37 °C in a humidified atmosphere containing 5% CO_2_. Subsequently, MTT solution (0.5 mg ml^−1^ in DMEM) was added to each well and incubated for 4 h. The medium was carefully aspirated, and the resulting formazan crystals were solubilized in dimethyl sulfoxide (DMSO). Absorbance was recorded at 595 nm using a microplate reader. Cell viability was expressed as a percentage relative to untreated controls and plotted as a function of nanoparticle concentration.

### 2.5. Radio frequency experimentation

The hyperthermia efficacy of Fe_3_O_4_ NPs was rigorously evaluated through a series of experiments utilizing short wave diathermy (SWD). SWD involves the use of high-frequency electromagnetic waves to generate heat within a targeted area, which is particularly effective for therapeutic purposes such as hyperthermia treatment for cancer. The primary goal of the study was to assess the ability of Fe_3_O_4_ to absorb and convert energy from an alternating magnetic field into heat, thus enabling the generation of localized heat for hyperthermic treatment. To initiate the experiment, first the NPs were dispersed in a physiological saline solution, which serves as a standard medium for *in vivo* applications, particularly in thermal therapies. Distilled water was utilized as the solvent to ensure proper nanoparticle suspension while preserving their stability and structural integrity throughout the process.

Two solutions were prepared:

• Test solution: contained 0.02 g of Fe_3_O_4_ NPs dispersed in 20 ml of physiological saline solution. The mixture was stirred using a magnetic stirrer for 10 minutes to achieve uniform dispersion before conducting the radiofrequency (RF) experiment.

• Control solution: prepared similarly, but without the addition of Fe_3_O_4_ to serve as a baseline for comparison.

The experiment was conducted using a SWD unit operating at a frequency of 27.12 MHz, commonly used in diathermy applications due to its deep tissue penetration and ability to interact with materials at the nanoscale. The unit could generate RF power levels ranging from 0 to 500 W cm^−2^, corresponding to RF intensity levels from 0 to 5. Both the test and control solutions were placed between RF electrode pads under identical experimental conditions to ensure accurate comparison. During the experiments, temperature changes within the system were closely monitored and recorded using infrared thermal imaging. This non-invasive technique provided real-time, high-resolution temperature data across the sample, enabling the precise measurement of thermal profiles. The infrared imaging allowed for the observation of both the spatial distribution of temperature within the sample and the temporal evolution of heat generation, which is crucial for understanding the efficacy of the NPs in a therapeutic context. The temperature profiles were recorded for every 5-minute intervals using a high-resolution FLIR E6 infrared thermal imaging camera.

#### 2.5.1 Coil parameters

For the magnetic hyperthermia experiments, the coil specifications of the modified shortwave diathermy (SWD) unit were as follows: field amplitude (*H*) = 24.8 kA m^−1^, operating frequency (*f*) = 265 kHz, and coil diameter = 55 mm. The calculated compliance factor (*H* × *f*) was approximately 6.6 × 10^9^ A m^−1^ s^−1^, which lies within or marginally above the generally accepted biological safety threshold. While conventional SWD devices typically operate at 27.12 MHz, the present study employed a lower-frequency alternating magnetic field (AMF) configuration, optimized for efficient heating of magnetic NPs in hyperthermia applications.

#### 2.5.2 SAR computation

SAR was calculated according to ISO/TS 19807-1 using the following expression: 
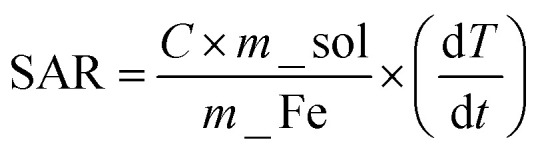
, where: *C* = 4.18 J g^−1^ °C^−1^ (specific heat of water), *m*_sol = 1 g (mass of solution), *m*_Fe = 0.005 g (mass of iron content in the NP dispersion), d*T*/d*t* = initial slope of temperature rise (°C min^−1^) converted to °C s^−1^, shown in Tables 1 and 4.

**Table 1:** Results of SAR analysis

Sample	d*T*/d*t* (°C min^−1^)	d*T*/d*t* (°C s^−1^)	SAR (W g^−1^)
2Y	0.81	0.0135	≈112.86
10Y	0.94	0.0157	≈131.25
18Y	0.76	0.0127	≈106.17

The derived SAR values demonstrate that 10Y exhibited the highest specific loss power can be attributed to factors like optimized particle size distribution, crystallinity and magnetic responsiveness. The heating efficacy was further validated through real-time infrared thermal imaging and was shown to scale linearly with field intensity (*R*^2^ = 0.88). All SAR values exceeded 100 W g^−1^, meeting or surpassing thresholds considered viable for achieving therapeutic hyperthermic temperatures (41–45 °C) under clinically relevant AMF exposure levels. These results are notably higher than many previous reports. For instance, Castellanos-Rubio *et al.* (2021) synthesized Fe_3_O_4_ NPs with SAR values of ∼50–100 W g^−1^, achieving ∼42–45 °C in 20–30 min under 30–100 kA m^−1^ fields at 100–500 kHz.^37^ Wei *et al.* (2012) reported a temperature rise to ∼40 °C in 25 min for 10–20 nm Fe_3_O_4_ NPs in aqueous dispersions.^38^ Arshad *et al.* (2018) demonstrated that Fe_3_O_4_ heating efficiency is strongly influenced by surface coating and dispersion medium, with significant degradation in saline environments due to aggregation.^39^ In contrast, the present study's biotemplated NPs maintain high SAR values even in saline dispersions, highlighting their potential as potent candidates for magnetically triggered cancer therapy.

## 3. Results and discussion

**Fig. 1:**
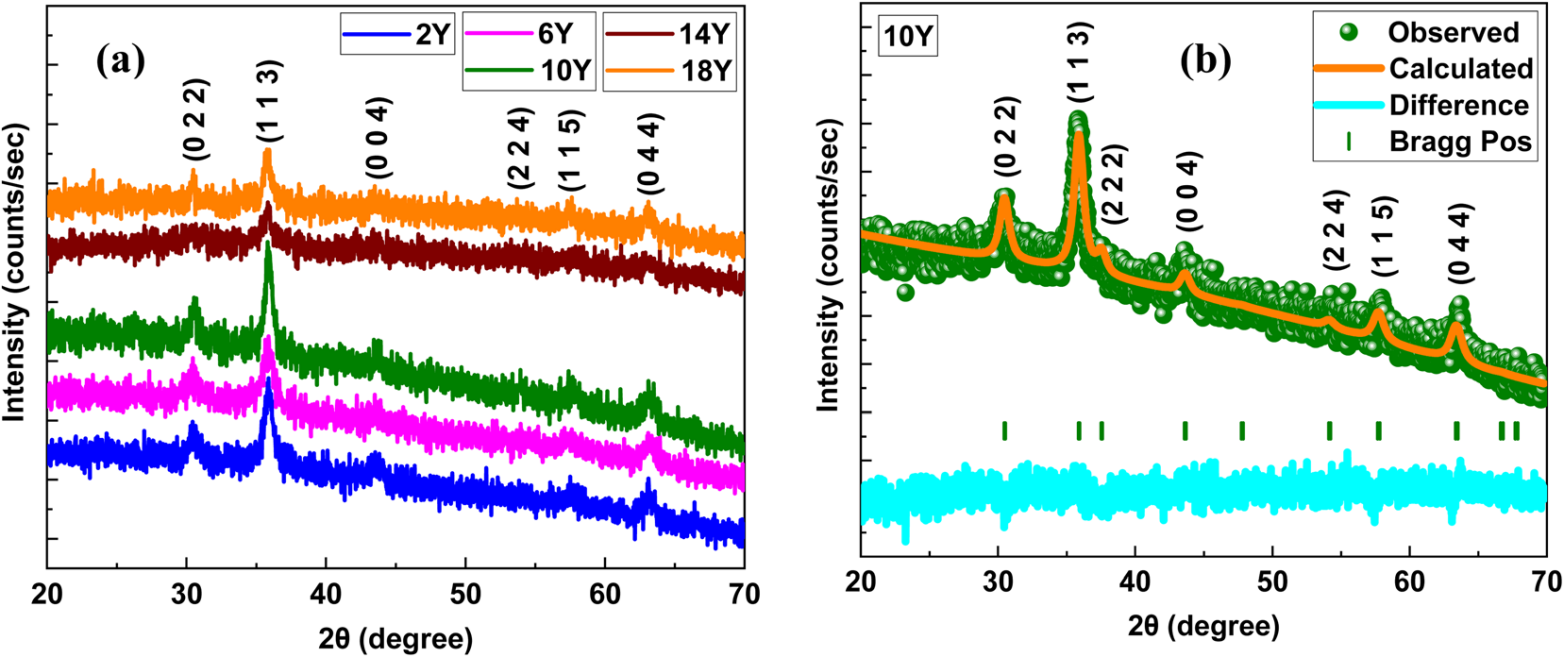
(a) XRD patterns of 2Y, 6Y, 10Y, 14Y and 18Y (b) Rietveld refinement of 10Y.

The synthesized Fe_3_O_4_ NPs were analysed using XRD to identify the crystal phase and degree of crystallinity. The recorded XRD patterns were plotted and shown in Fig. 1 where Fig. 1(a) shows the XRD patterns of 2Y, 6Y, 10Y, 14Y, 18Y. As a representative of the series, Rietveld refined XRD profile of 10Y sample is shown in Fig. 1(b). It is observed that all the samples are crystallized into a phase of Fe_3_O_4_. Among all the samples, 2Y and 10Y exhibited significantly good crystallinity. The major diffraction peaks at 30.529, 35.81, 43.42, 53.961, 57.382, and 63.119 corresponding to the (0 2 2), (1 1 3), (0 0 4), (2 2 4), (1 1 5), and (0 4 4) planes of Fe_3_O_4_ (ICSD Ref Code: 98-011-1046).^38–41^ All the XRD peaks of the samples are ascribed to the phase of cubic type and with the space group of *Fd*3̄*m*.^42,43^ The refined parameters obtained through refinement can include lattice parameters, atomic positions, occupancy factors, and other crystallographic properties with *R*-factor (Good fit) are listed in the following Table 2.

**Table 2:** Rietveld refinement data of 10Y

		Cell parameters					Site occupation		
Structure	Cubic	*a*	8.2475	O1	*x*	0.25510	O1	1.00000	
Space group	*Fd*3̄*m*	*b*	8.2475		*y*	0.25510	FE1	1.00000	
Volume (Å^3^)	561.0023	*c*	8.2475		*z*	0.25510	FE2	1.00000	
Density (g cm^−3^)	12.095	*α*	90°	FE1	*x*	0.50000	**Agreement factors**		
		*β*	90°		*y*	0.50000	*R*_p_	18	
		*γ*	90°		*z*	0.50000	*R*_wp_	15.7	
				FE2	*x*	0.12500	*R*_e_	10.9	
					*y*	0.12500	Chi^2^	1.162	
					*z*	0.12500			

**Fig. 2:**
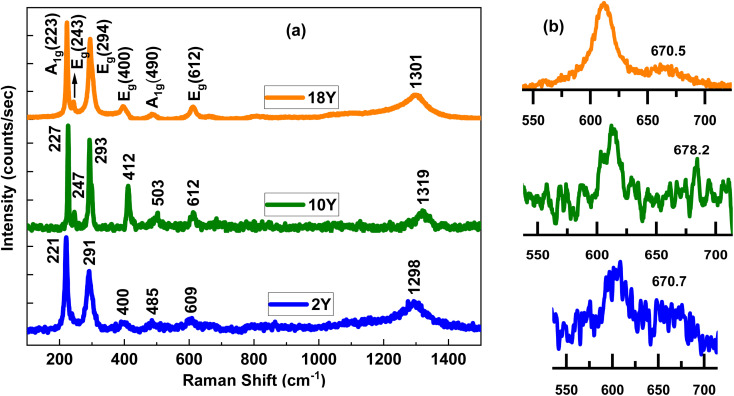
(a) Raman spectrum of 2Y, 10Y and 18Y (b) enlarged view at ∼670 cm^−1^.

In order to gain deeper insights into the structural information, the samples 2Y, 10Y and 18Y were subjected to RS analysis and are depicted in Fig. 2. RS was performed to identify the vibrational and rotational bands specific to the synthesized material. Several Raman spectroscopic studies of Fe_3_O_4_ have been conducted, and the characteristic bands of Fe_3_O_4_ are typically located at approximately 670, 538, and 306 cm^−1^.^27,31,32^Fig. 2(a) presents the Raman spectra of the synthesized Fe_3_O_4_ NPs. However, most of the observed peaks correspond to Fe_2_O_3_, indicating that Fe_3_O_4,_ underwent a partial oxidation during Raman the scattering. This oxidation is attributed due to the thermal effects of high laser power, which could induce a localized increase in temperature, leading to the conversion of Fe_3_O_4_ (magnetite) to Fe_2_O_3_ (hematite). The oxidation process is particularly observed in nanoscale range materials, where surface energy and defect sites facilitate phase transformation under external stimuli.^44–47^ Despite this, a small broad peak can be seen in the range of 670–680 cm^−1^ in all the synthesized samples, which corresponds to the major A_1g_ vibrational mode of Fe_3_O_4_, associated with symmetric Fe–O stretching.^48^ The 2Y, 10Y, and 18Y samples exhibit broad peaks at 670.7, 678.2, and 670.5 cm^−1^, respectively, confirming the presence of Fe_3_O_4_. An enlarged view of the Fe_3_O_4_ major peak for all samples is shown in Fig. 2(b).

**Fig. 3:**
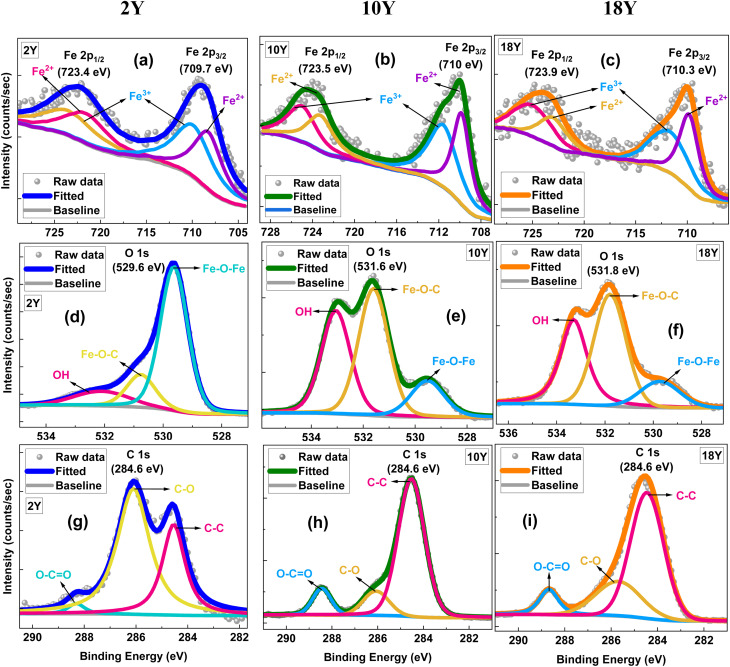
XPS analysis of (a), (b) and (c) Fe 2p of 2Y, 10Y and 18Y. (d), (e) and (f) O 1s of 2Y, 10Y and 18Y. (g), (h) and (i) C 1s of 2Y, 10Y and 18Y respectively.

To further characterize the surface chemical composition of the 2Y, 10Y and 18Y samples, XPS analyses were conducted. Fig. 3 with each column shows the Fe 2p, O 1s, C 1s spectrum for 2Y, 10Y and 18Y respectively. The wide spectrum for all the samples is displayed in the supplementary information Fig. SF1. The XPS profile demonstrates that the peaks of Fe 2p, O 1s, and C 1s were observed in the hybrids, and no other elemental peak is detected, which reveal that synthesized samples mainly contained three elements of Fe, O, and C and avoid of any impurities. In the fitted Fe 2p spectrum of all the samples, there are two broad peaks at ∼709.7 eV, ∼723.4 eV (2Y), ∼710 eV, ∼723.5 eV (10Y), and at ∼710.3 eV, ∼723.9 eV (18Y), corresponding to Fe 2p_3/2_ and Fe 2p_1/2_ respectively shown in Fig. 3(a)–(c), ascribed to spin–orbit coupling which agreed well with the previous reports of Fe_3_O_4_.^48–50^ The absence of satellite peaks in the Fe 2p spectrum of XPS, which is a characteristic feature of confirmation of Fe_3_O_4_ structure.^51^ Each Fe 2p peak comprises of deconvoluted peaks of trivalent Fe^3+^ and divalent Fe^2+^ oxidation states of Fe_3_O_4_. The Fe^3+^/Fe^2+^ quantitative ratios for 2Y, 10Y, and 18Y are 1.36, 1.12, and 1.19 respectively. The deconvoluted peaks of the O 1s XPS spectrum in Fig. 3(d)–(f) are Fe–O–Fe, Fe–O–C, OH indicating the linkage of Fe_3_O_4_ with O.^52–54^ The C 1s XPS spectrum of the samples in Fig. 3(g), (h) and (i) exhibits three major peaks with binding energies at 283 to 285 eV, which were consistent with the C=C, C–OH, and C=O configuration.^48–50,54–56^ It is observed that in C 1s peaks, C–C bond intensity is higher than C–O in 10Y and 18Y, which is attributed to the increased carbon content,^57–62^ which results from the larger amount of stabilizing agent that converts into carbon during calcination at 400 °C. Additionally, analysis of the O 1s peaks reveals that 2Y exhibits a lower intensity Fe–O–C peak, whereas 10Y and 18Y display higher intensity Fe–O–C peaks, indicating stronger carbon linkages. Overall, XPS analysis of the C 1s and O 1s peaks confirms that an increase in the stabilizing agent leads to a corresponding rise in carbon content.

**Fig. 4:**
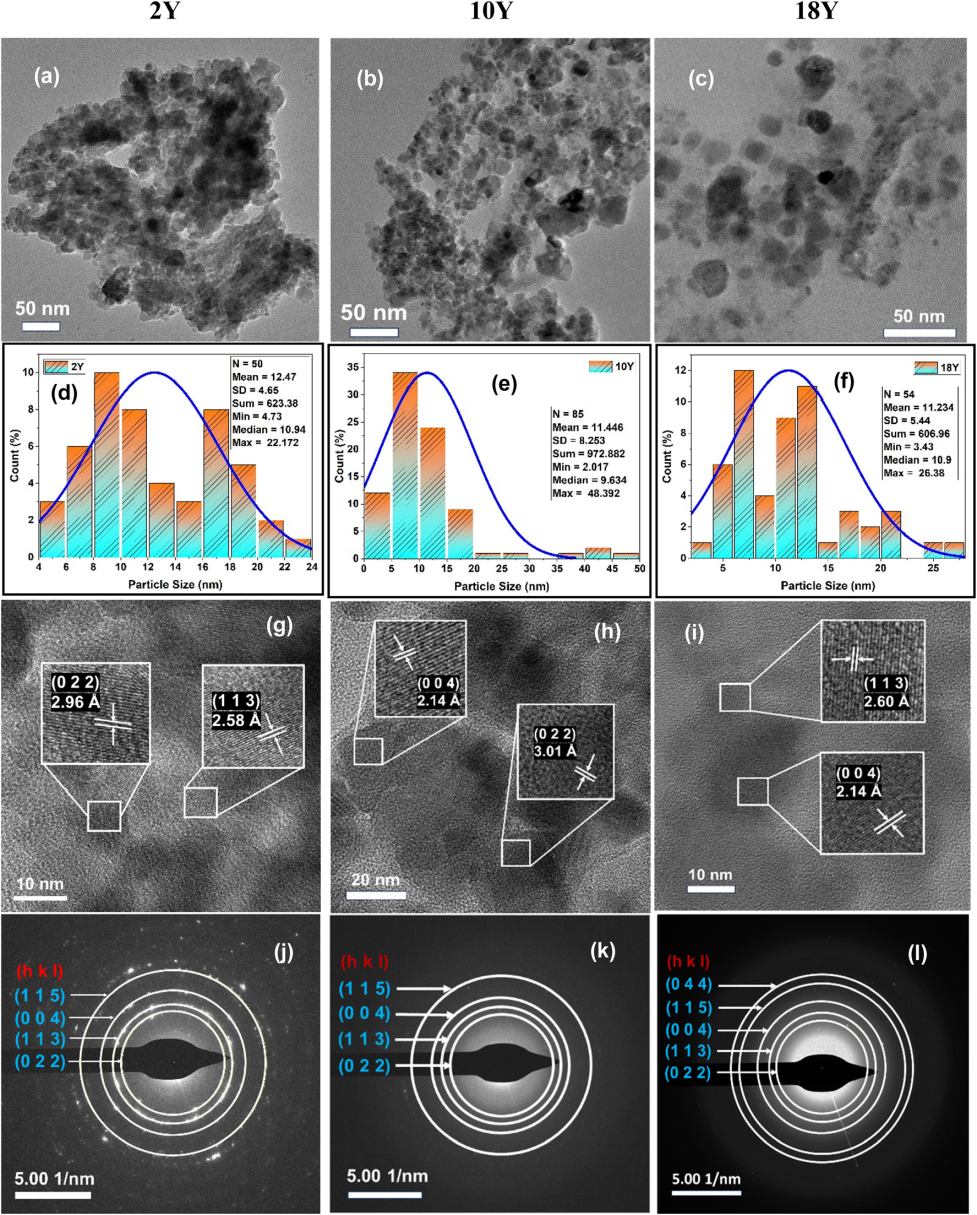
HRTEM. (a)–(c) HRTEM of 2Y, 10Y and 18Y. (d)–(f) Particle size distribution of 2Y, 10Y and 18Y. (g)–(i) Lattice spacing of 2Y, 10Y and 18Y. (j), (k) and (l) SAED pattern of 2Y, 10Y and 18Y respectively.

The microstructure and morphology were also analysed through HR-TEM and selected area electron diffraction (SAED) patterns for all the samples as shown in Fig. 4. The HR-TEM images of all the samples were observed to be in polygonal shaped with particle sizes varying from 10–30 nm range shown in Fig. 4(a)–(c). The particles are observed to be agglomerated in case of 2Y sample where as well-defined particles are noticed in 18Y sample, suggesting the increase in yolk concentration while synthesizing is resulting in well-defined particles. It can also be possible to get a small layer of carbon on each Fe_3_O_4_ NPs that makes the particles are separated in the case of higher concentrated yolk samples. The particle size distributions of all the samples are shown in Fig. 4(d)–(f). The interplanar spacing between two lattice planes is calculated and that corresponds to the respective plane of all the samples are shown in Fig. 4(g)–(i). The interplanar spacing between two lattice planesis determined through ImageJ software, with the identified spacing used to indicate the respective plane within the lattice structure. The SAED images in Fig. 4(j)–(l) clearly show that the lattice spacing without any contortion, designating the NPs with high purity. The planes in the SAED images are matching with that of the planes obtained in the XRD diffraction peaks.

**Fig. 5:**
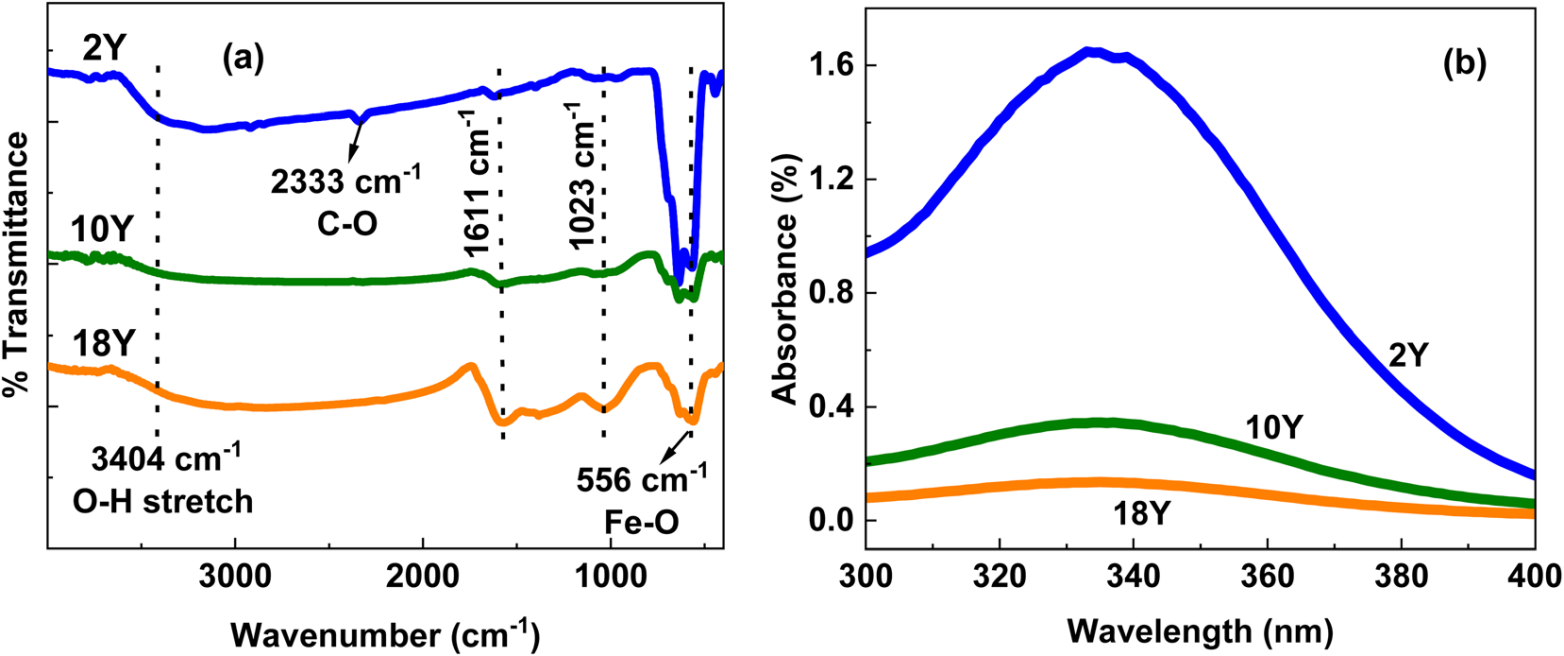
(a) FTIR (b) UV of 2Y, 10Y and 18Y.

The Fig. 5(a) shows the FTIR analysis of all the samples. A broad peak at 3404 cm^−1^ represents the O–H stretching, peaks at 1611 cm^−1^ are ascribed to the vibrations of C=O. The two dominant vibration bands are observed in the 400–600 cm^−1^ region of FT-IR spectra peaks in all the samples can be ascribed to the bending vibration of O–Fe–O bond and stretching vibration of the Fe–O bond respectively.^19,29,63–66^ The complete disappearance of the multiple sharp deutoplasm-related peaks^67^ in the 1650 to 1750 cm^−1^ region after calcination confirms the elimination of potential immunogenicity concerns.

The UV-visible spectra analyses of all the samples are shown in Fig. 5(b) and the respective tauc plots are displayed in supplementary information in Fig. SF2. It shows an intense absorption between ∼300 to ∼400 nm with an absorption coefficient that gradually decreases from ∼340 nm with increasing wavenumber. The absorbance 2Y is higher compared to other samples, whereas all other samples exhibit a trend of decreased absorbance with higher concentration of deutoplasm. The band gap energy of samples 2Y, 10Y and 18Y is 2.91 eV, 2.86 eV, 2.84 eV respectively and is determined using Tauc's relation, (*αhν*)^1/*γ*^ = *B*(*hν* − *E*_g_) where, *γ* = 1/2 or 2 for direct or indirect respectively.^22,29,63,68,69^

**Fig. 6:**
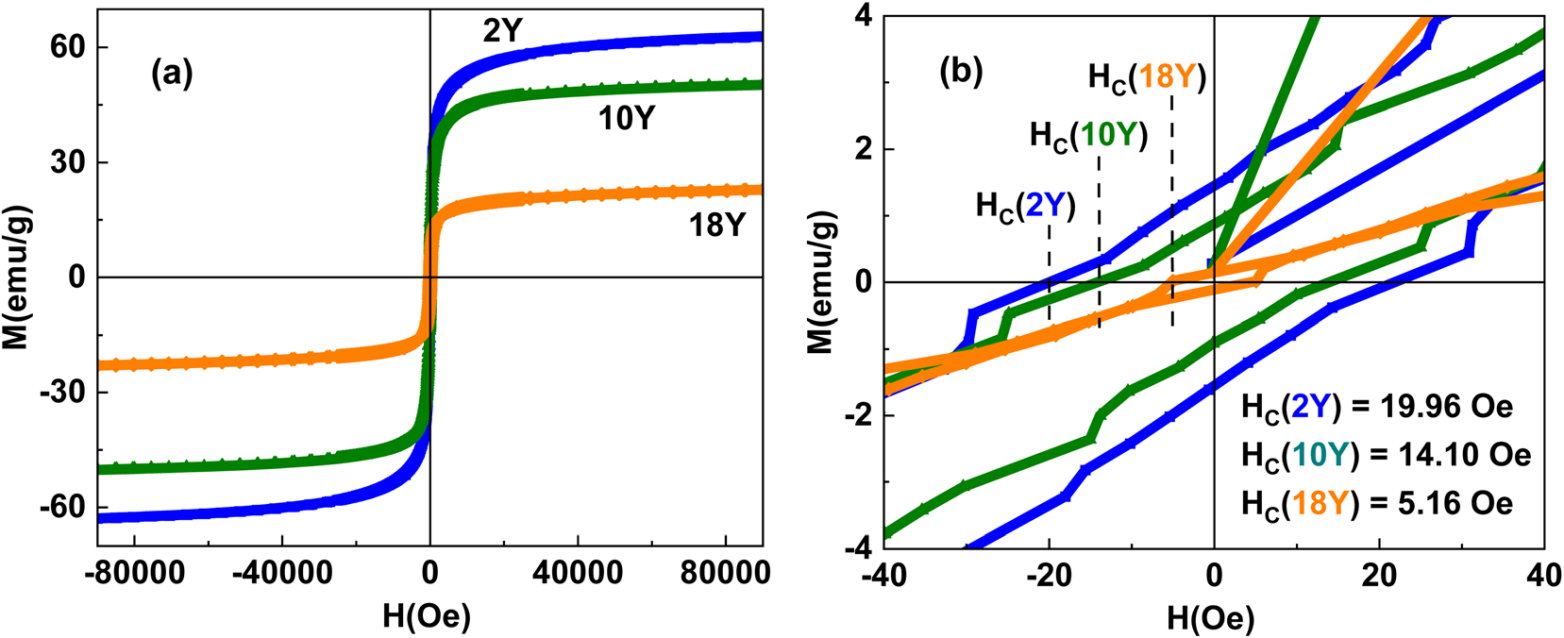
(a) M–H curve (b) closure view of M–H curve.

Magnetic behaviour of all the samples was studied at room temperature in order to understand the response of the samples under the influence of magnetic fields. Field variation of magnetization in the form of hysteresis plots was recorded at 300 K, and depicted in Fig. 6(a). It is noticed that all the samples displayed an S-shaped curves with clear hysteresis at lower applied magnetic fields. The closure pictures of the hysteresis curves are visible and displayed in the Fig. 6(b). The coercivity (*H*_c_) of 2Y, 10Y and 18Y was obtained as 19.96 Oe, 14.10 Oe and 5.16 Oe, which is systematic decrease in their *H*_c_ values with the increase of yolk-concentration. The magnetization plots show a sharp rise in their initial magnetisation curves coupled with a non-saturating tendency even at higher fields of 9 T. Saturation magnetisation (*M*_s_) values of all the samples were determined by using extrapolation methods, *i.e.*, extrapolating *x*-axis from high fields to 1/*H* = 0 from *M vs.* 1/*H* plot. The *M*_s_ values estimated for 2Y, 10Y, 18Y was 57.6 emu g^−1^, 46.83 emu g^−1^ and 20.54 emu g^−1^ respectively, which is again decreasing systematically with the increase of yolk-concentration. The presence of an S-shaped hysteresis loops is suggestive of a superparamagnetic (SPM)-like behaviour. Again, a sharp rise in initial magnetization curve at lower magnetic fields coupled with finite *H*_c_ values are indication of soft ferromagnetic (FM) behaviour. Ferromagnetism (FM) is characterized by long-range magnetic ordering, where individual magnetic moments align parallel even in the absence of an external field, leading to finite coercivity (*H*_c_) and remanence (*M*_r_). In contrast, superparamagnetism (SPM) is typically observed in nanosized single-domain particles, where thermal energy is sufficient to randomly flip magnetic moments, resulting in zero or negligible *H*_c_ and *M*_r_. These findings collectively suggests the coexistence of FM and SPM-like nature of Fe_3_O_4_ NPs, which are in agreement with the earlier reports.^2,38,48,68,70–72^ The systematic decrease in the magnetic behaviour of Fe_3_O_4_ NPs with increasing yolk concentration during synthesis can be attributed to the formation of a thin carbonaceous layer on the particle surface. This non-magnetic carbon layer acts as a barrier, reducing the effective magnetic volume and weakening interparticle exchange interactions.^7,8^ As a result, the saturation magnetisation (*M*_s_) decreases progressively from 57.6 emu g^−1^ for 2Y to 20.54 emu g^−1^ for 18Y, which is consistent with the HRTEM observations and the C 1s spectra obtained from XPS analysis.

### 3.1. Cytotoxicity analysis

**Fig. 7:**
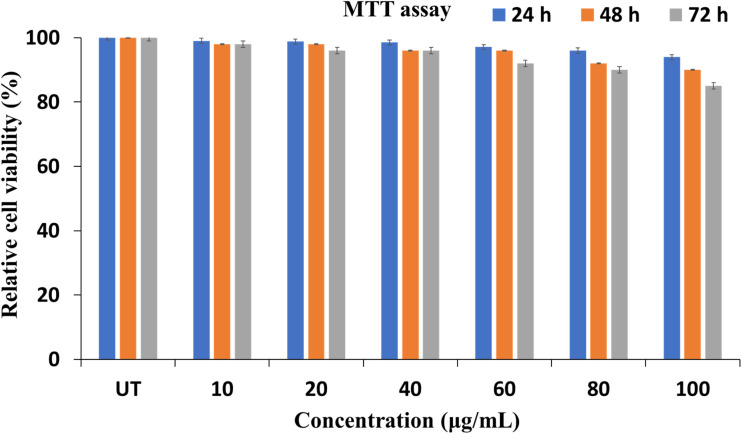
Cell viability (%) of HEK293 cells for 24, 48, and 72 hours.

The cytotoxicity assessment was carried out only for the 2Y sample, as all the samples were synthesized using the same stabilizing agent. The results revealed that the 2Y NPs exhibited high biocompatibility toward HEK293 cells. Across all tested concentrations (10–100 μg ml^−1^) and incubation periods (24, 48, and 72 h), cell viability remained above 80%. Notably, even at the highest concentration of 100 μg ml^−1^, viability was approximately 85%, indicating minimal cytotoxic effects and supporting the suitability of the nanoparticles for potential biomedical applications. The cell viability (%) of HEK293 cells was evaluated after exposure to varying concentrations of the formulated nanoparticles (10–100 μg ml^−1^) for 24, 48, and 72 h, as shown in Fig. 7.

### 3.2. Hyperthermia applications of Fe_3_O_4_ NPs

**Fig. 8:**
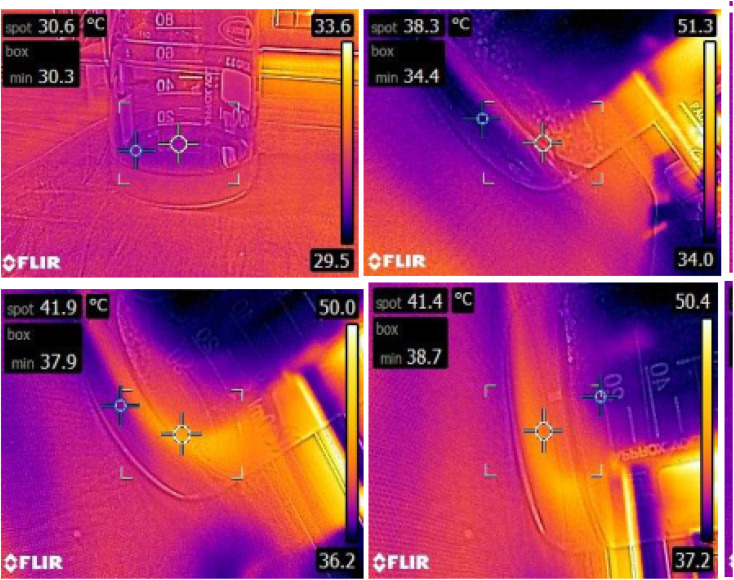
Thermal images of control solution and 2Y test solution.

In order to study the heating ability of the developed Fe_3_O_4_ NPs, RF experimentation was performed on all the samples. It is understood that the higher magnetization values are capable of producing maximum heating ability, and thus we have carried out the radiofrequency experiments on 2Y sample. Thermal images of both the control solution (first row) and 2Y test solution (second row) were captured on 2Y sample to provide temperature mapping at various points and the same is shown in Fig. 8. The thermal mapping revealed significantly higher temperatures in the test solution compared to the control. The test solution reached a peak temperature of 41.9 °C, which is within the therapeutic range for RF hyperthermia-based cancer therapy.

Subsequent experiments on the 2Y test solution were conducted at varying RF intensities (0–4), with corresponding power levels ranging from 0 to 500 W cm^−2^ with levels mentioned as 0–4. Temperature profiles were recorded over time to study the heating behaviour of the Fe_3_O_4_ NPs. The temperature profiles of the 2Y nanoparticle dispersions revealed a gradual increase in temperature upon exposure to the alternating magnetic field. As the electromagnetic field interacted with the NPs, a steady rise in temperature was observed, reaching a peak temperature range of 26.0 °C to 42.0 °C in just 30 minutes of span. This range is significant for hyperthermic treatments, as temperatures between 41 °C and 45 °C are typically required for effective therapeutic outcomes, such as selective tumour cell destruction or enhanced drug delivery *via* heat-induced permeability changes in cell membranes. The temperature profiles revealed that, as the intensity of the applied alternating magnetic field increased, the temperature of both distilled water and nanoparticle dispersions rose steadily. This is consistent with the fundamental principles of electromagnetic energy absorption, where higher field intensities facilitate greater energy absorption and subsequent heating of the medium. The temperature increases in both cases was relatively smooth, indicating a direct relationship between magnetic field intensity and thermal output. This trend suggests that increasing the intensity of the electromagnetic field can be an effective method for enhancing the heating potential, regardless of the medium's composition, albeit with distinctions in the ultimate temperature levels reached.

**Fig. 9:**
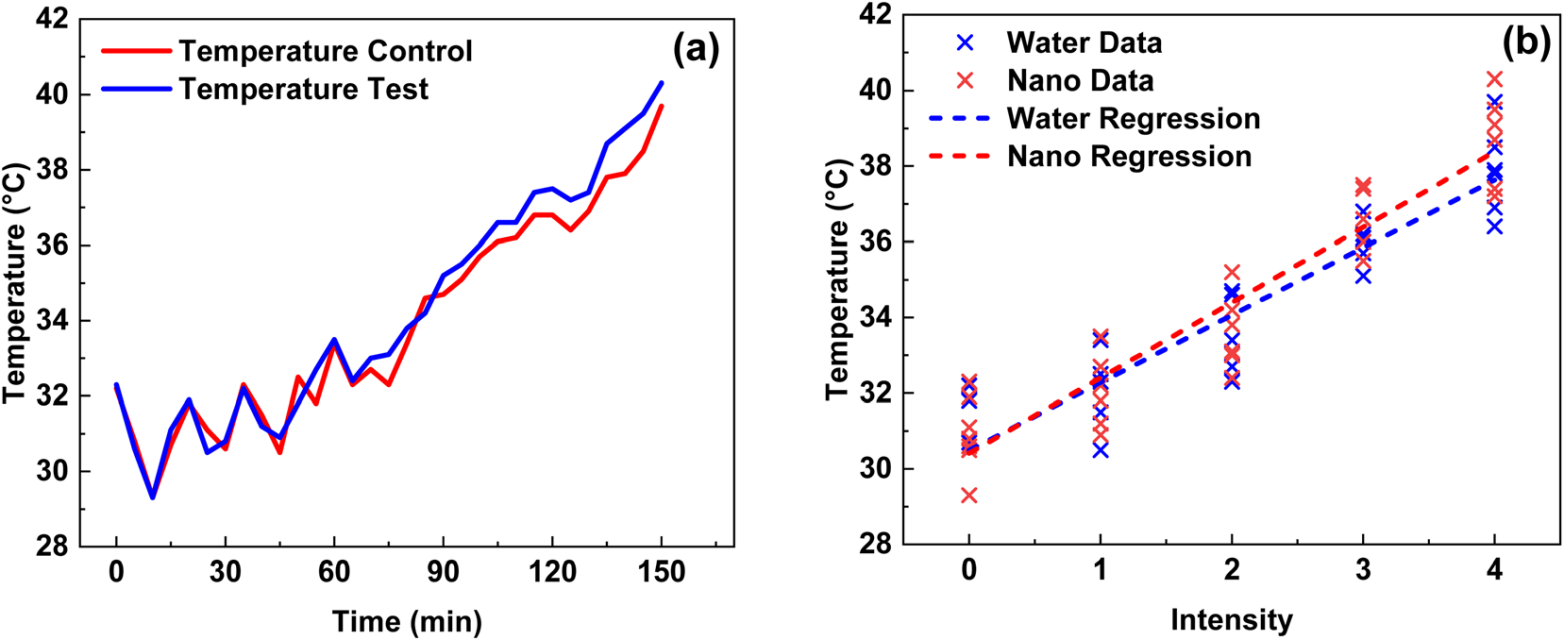
(a) Temperature *vs.* time under the RF exposure of 2Y (b) linear regression curve of test solution of 2Y.

A more nuanced observation was the difference in temperature between the two mediums. The temperature profiles shown in Fig. 9(a) revealed that, while both the distilled water and nanoparticle dispersions exhibited a similar initial rate of increase, the blue curve (representing the NPs) slightly exceeded the red curve (representing distilled water) over time. This indicates that the 2Y NPs exhibited superior heat retention properties compared to distilled water. The slightly higher temperature reached by the nanoparticle dispersion suggests that the 2Y NPs may have a higher specific heat capacity or enhanced ability to absorb and retain heat, leading to more efficient thermal retention.

This enhanced heat retention could be attributed to several factors inherent in the NPs properties. NPs, especially those designed for hyperthermia applications, often possess specific characteristics such as increased surface area and magnetic responsiveness, which can facilitate the more efficient absorption and conversion of electromagnetic energy into heat. Additionally, the unique interactions between the NPs and the alternating magnetic field may lead to localized heating effects, further contributing to the temperature difference observed between the two media. These interactions could include phenomena like magnetic dipole alignment and relaxation processes, which are known to generate heat in magnetic materials.

From the above observation, the witnessed temperature trends and differences between the distilled water and nanoparticle dispersions highlight the promising potential of 2Y NPs for hyperthermia applications. While both media exhibit similar thermal trends with respect to field intensity, the NPs enhanced heat retention capabilities demonstrate their superiority in terms of achieving and maintaining the desired therapeutic temperature. This difference in thermal behaviour reinforces the potential of 2Y NPs as an effective agent for localized hyperthermic treatment, where precise temperature control is crucial for therapeutic efficacy. Further studies focusing on optimizing the NPs properties and evaluating their performance at varying intensities and frequencies could provide deeper insights into their role in advancing hyperthermic therapies.

Regression analysis revealed a strong positive linear relationship between intensity and temperature as shown in Fig. 9(b) linear regression plot for temperature *vs.* intensity: Blue Line (Water Regression) shows a moderate increase in temperature with intensity whereas, Red Line (Nano Regression) exhibits a steeper slope, confirming better heat absorption by NPs. This visually supports our previous regression analysis, showing that NPs have a stronger heating effect. Both the test and control solutions showed increasing temperatures with higher RF intensities, indicating the crucial role of intensity in modulating the thermal response of this 2Y Fe_3_O_4_ NPs. Notably, the heating effect of the 2Y solution increased progressively with rising RF intensities. At the highest intensity level (4), it took approximately 30 minutes to reach the target temperature of 41–42 °C, which is sufficient for effective hyperthermia-based cancer treatment. Further, a statistical analysis of the of the temperature profiles of the control and test solutions of 2Y was done (as mentioned in Table 3) and it is proved that the 2Y Fe_3_O_4_ NPs are significantly has higher temperature than the control solution with coefficient of 0.94 with *p* < 0.001. Hence 2Y is proved to be potential candidates for the hyperthermia application of cancer therapy.

**Table 3:** Statistical analysis of the temperature profiles

Metric	Temperature control	Temperature test
Correlation (*r*)	0.93	0.94
Regression equation	Intensity = −14.58 + 0.49 × temperature_control	Intensity = −13.29 + 0.44 × temperature_test
*R* ^2^	0.87	0.88
*P*	0.0000	0.0000
Statistical significance	Highly significant (*p* < 0.001)	Highly significant (*p* < 0.001)

**Fig. 10:**
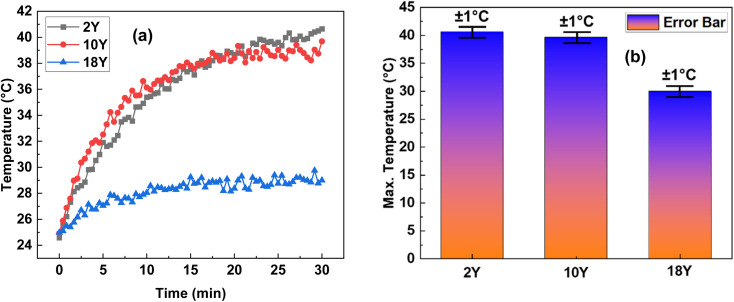
(a) Temperature profiles for 2Y, 10Y, and 18Y NP solutions under RF exposure (b) error bars represent ±1 standard deviation from three independent experimental trials.

It is worthy to mention here that, the experiments were also included control tests with a saline solution alone, without the presence of NPs. The results from these control experiments demonstrated a significant temperature rise, but not to the extent observed in the presence of 2Y NPs. This suggests that the saline solution itself was minimally responsive to the electromagnetic field at the frequency of 27.12 MHz. The key observation here was that the magnetic frequency of 27.12 MHz was not sufficient on its own to induce significant heating in the 2Y NPs, indicating that the NPs themselves play a critical role in the thermal response. Following the initial experimentation with 2Y NPs, the study was extended to investigate the hyperthermia effects of two additional samples: 10Y and 18Y. Similar results were observed for 10Y, which exhibited faster heating than 2Y but did not reach as high as 2Y. In contrast, 18Y did not achieve the expected temperature rise. The temperature profiles for all three samples were recorded over time under a constant RF intensity of 4 and are presented in Fig. 10. The error bars shown in the Fig. 10(b) represent ±1 standard deviation, calculated from three independent experimental trials. This statistical representation captures the variability within the dataset and reflects the reproducibility of the measurements across separate runs, providing confidence in the reliability of the observed trends.

The comparison reveals distinct differences in the thermal performance of the three samples and shown in Table 4. The study found that 2Y provides steady and reliable heating, making it ideal for controlled hyperthermia. 10Y exhibits moderate heating, which might be useful for milder thermal applications. 18Y delivers the most rapid and intense heating, suitable for short-duration, high-intensity treatments.

**Table 4:** Comparison of the temperature profiles of 2Y, 10Y and 18Y

Sample	Count	Avg. temperature (°C)	Min temp (°C)	Max temp (°C)	Std dev (°C)	Observation
2Y	23	36.90	29.3	41.6	3.30	Moderate temperature with some variations
10Y	42	28.06	26.3	29.5	0.85	Lowest and most stable temperature
18Y	66	34.79	25.8	39.2	3.70	Highest variation in temperature

This outcome highlights the importance of the nanoparticle's physical and chemical properties in determining its ability to absorb and convert RF energy into heat. It suggests that, despite the presence of a suitable magnetic field, the NPs may require additional factors such as size, surface coating, or composition to optimize their interaction with the electromagnetic field for effective hyperthermic treatment. The magnetic properties of the NPs, such as their magnetic susceptibility and heat generation capacity, are key factors that determine the efficiency of energy conversion and the subsequent thermal effect. Therefore, while the initial experiments revealed that synthesized nanoparticles could generate heat when exposed to a 27.12 MHz electromagnetic field, the temperature increase was insufficient without additional factors to enhance the heating capability of the nanoparticles. This indicates that further modifications to the nanoparticle design or the use of higher magnetic field intensities or frequencies may be required to achieve more effective thermal responses for therapeutic purposes. These findings underscore the complex interplay between nanoparticle characteristics, electromagnetic field parameters, and the resulting thermal effects, which must be optimized for successful hyperthermia treatments. Future studies could explore the use of different nanoparticle compositions, sizes, or surface functionalization's to enhance their ability to convert RF energy into heat, thus improving their potential for clinical hyperthermia applications.

In the experiments, both distilled water (saline medium) and the synthesized nanoparticle dispersions exhibited similar temperature trends over time. This observation suggests that, at a fundamental level, both media exhibit a comparable thermal response to the application of an alternating magnetic field. Initially, the temperature of both the distilled water and nanoparticle solutions increased gradually as the electromagnetic energy was absorbed. However, while their general thermal progression followed similar patterns, the key differentiator became evident when examining the rate of temperature rise and the final temperatures achieved. This heating in the control saline medium occurs mainly through dielectric polarization and ionic conduction under the RF field, while NPs generate heat *via* magnetic relaxation, enabling more efficient RF to heat conversion. Despite similar overall trends, nanoparticle dispersions showed faster temperature rise and higher final temperatures, highlighting the influence of their magnetic properties on hyperthermia performance. The analysis of the Fe_3_O_4_ NPs reveals a strong positive linear relationship between intensity and temperature. Both temperature control and test conditions significantly affect the intensity, with similar regression coefficients and high correlation values.

This study provides valuable insights into the behaviour of nanomaterials in response to temperature changes, which could be crucial for further research and applications in nanotechnology and material science. The comparative study with previous reports is shown in Table 5.

**Table 5:** Comparative study with previous reports

Study	Nanoparticle type	Key findings
Our study	2Y, 10Y, 18Y	• Nanoparticles exhibit higher temperature retention compared to distilled water
		• Show significant heating effects
Mansfield *et al.*^73^	Various nanoparticles	• Thermal analysis methods provide insights into particle composition and crystallinity
		• Techniques like thermogravimetric analysis and differential scanning calorimetry are essential for nanoparticle characterization
Yi *et al.*^74^	Charged nanoparticles	• Nanoparticle charge and temperature significantly influence thermophysical properties
		• Interaction between charged nanoparticles and solvents affects viscosity and thermal conductivity
Xie & Qin *et al.*^75^	Nanoparticle arrays	• Developed analytical solutions for transient heating of nanoparticle arrays
		• Proposed the concept of thermal resolution to quantify heating dynamics
Roodbari *et al.*^76^	TiO_2_ nanoparticles	• Investigated interfacial thermal conductance between TiO_2_ nanoparticles and water
		• Found that Kapitza conductance of TiO_2_ is higher than other conventional nanoparticles
Donovan *et al.*^77^	Silica nanoparticles with polymer bridging	• Explored thermal transport in disordered packings of amorphous nanoparticles
		• Found that interstitial polymer eliminates boundary scattering, increasing overall thermal conductivity
Tielke & Avila *et al.*^78^	Various nanoparticles in ethylene glycol	• Conducted statistical analysis of thermal conductivity in nanofluids
		• Found that thermal conductivity increases linearly with concentration; nanoparticle size significantly influences results for alumina and titania

This study provides valuable insights into the behaviour of nanomaterials in response to temperature changes, which could be crucial for further research and applications in nanotechnology and material science. Our findings align with previous studies indicating that nanoparticles enhance thermal properties compared to base fluids.^53–59^ The minimal heating effect observed in 2Y highlights the importance of nanoparticle composition, suggesting that not all nanoparticles equally enhance thermal. While our study focused on empirical temperature measurements, other studies employed techniques like molecular dynamics simulations and statistical analyses to explore thermal behaviour. This comparative analysis underscores the significance of nanoparticle composition and the need for diverse methodological approaches to fully understand their thermal properties.

## 4. Conclusion

Fe_3_O_4_ nanoparticles were successfully synthesized *via* a simple, cost-effective sol–gel method using egg deutoplasm as the reaction medium. XRD analysis confirmed the formation of a pure cubic spinel structure, while morphological studies revealed well-defined nanoparticles in the size range of 10–30 nm. The particles exhibited a narrow optical band gap of 2.8–2.9 eV, indicative of favourable electronic properties. Cytotoxicity evaluation using the MTT assay confirmed excellent biocompatibility for the 2Y sample. Magnetic measurements showed the coexistence of ferromagnetic (FM) and superparamagnetic (SPM) behaviour, with a coercivity of 19.96 Oe and a saturation magnetization of 57.6 emu g^−1^. In RF hyperthermia studies, the 2Y Fe_3_O_4_ NPs synthesized with the lowest deutoplasm concentration (2Y) – achieved a maximum temperature of 41.6 °C, aligning with the optimal range for therapeutic hyperthermia. These NPs demonstrated rapid heating capability and good thermal retention, making them suitable for applications requiring either fast temperature elevation or sustained, controlled heating during long-duration treatments. Thus, the 2Y Fe_3_O_4_ NPs represent a promising intermediate option for moderate heating requirements in biomedical applications. Future investigations focusing on colloidal stability, *in vivo* biocompatibility, and long-term performance will further strengthen the clinical translation potential of these NPs for cancer hyperthermia therapy.

## Author contributions

Y. Ranjith Kumar: conceptualization, methodology, visualization, investigation, writing – original draft. Pragya Trivedi: formal analysis, investigation. Avijit Jana: formal analysis, investigation, validation. D. Suman: investigation, resources, writing – review & editing. M. Vasundhara: supervision, conceptualization, funding acquisition, resources, writing – review & editing.

## Conflicts of interest

The authors declare that they have no known competing financial interests or personal relationships that could appear to influence the work reported in this paper.

## Supplementary Material

RA-015-D5RA03372A-s001

## Data Availability

The authors confirm that the data supporting the findings of this study are available in the data repository and also can be made available on request. Supplementary information is available. See DOI: https://doi.org/10.1039/d5ra03372a.
