# Supplementary material for: Preparation of biotemplated Fe3O4 nanoparticles and evaluation of RF-induced heating efficiency for targeted hyperthermia
Source: RSC Adv. 2025 Oct 6;15(44):36879–94. doi: 10.1039/d5ra03372a (PMC12498135; doi:10.1039/d5ra03372a)
Supplement: RA-015-D5RA03372A-s001 [file RA-015-D5RA03372A-s001.pdf]

Supplementary Information (SI) for RSC Advances.
This journal is © The Royal Society of Chemistry 2025

Supplementary Information (SI):

XPS Wide Spectrum

The wide spectra of samples 2Y, 10Y and 18Y are displayed in the Figure SI1 (a), (b), and (c) respectively.

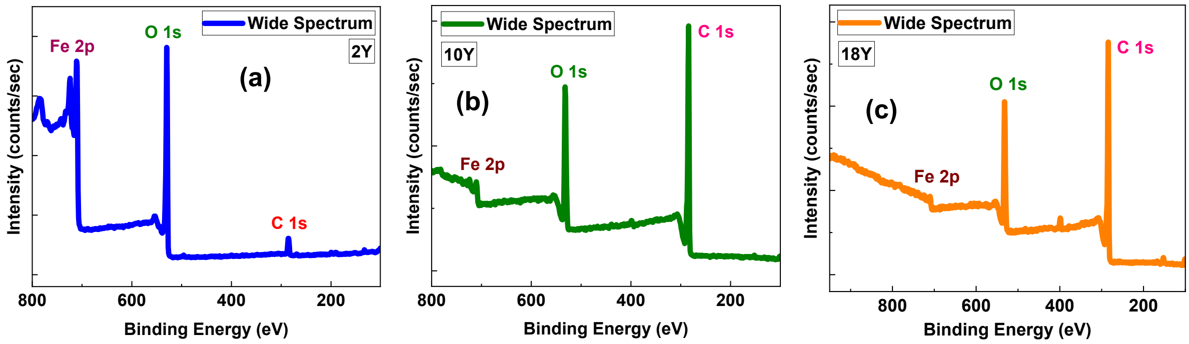

Figure SI1. Wide Spectrum of (a) 2Y (b) 10Y (c) 18Y

Tauc Plot

The band gaps are calculated using tauc's relation and are obtained as 2.91 eV, 2.86 eV, and 2.84 eV for 2Y, 10Y and 18Y as displayed in Figure SI2 (a), (b) and (c) respectively.

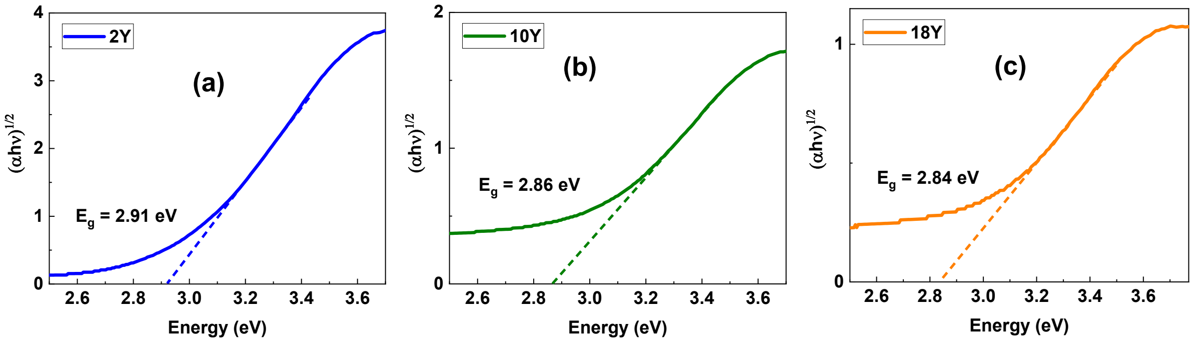

Figure SI2. Tauc plot of (a) 2Y (b) 10Y (c) 18Y

Repetetion of RF experiment N=3

The Radio Frequency experiment was performed in triplicate (N = 3) for each of the samples 2Y, 10Y, and 18Y, as shown in Figure SI3 (a–c). Among these, the most representative dataset was selected for presentation, and the corresponding error bars have been provided in the main manuscript.

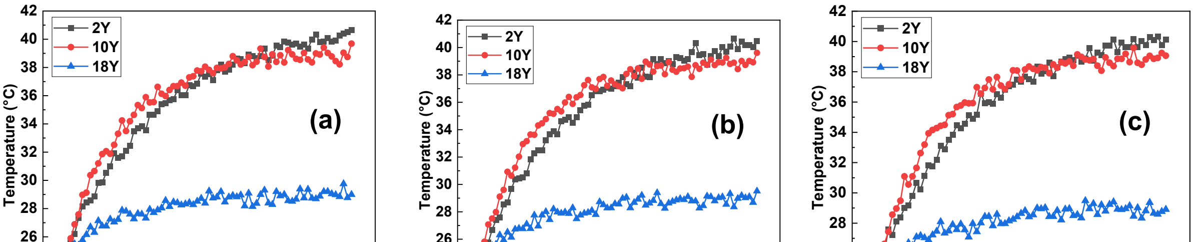

Figure SI3. The RF experiment is repeated 3 times (a) Experiment-1 (b) Experiment-2 (c) Experiment-3. The error bar is ±1

DLS Measurements

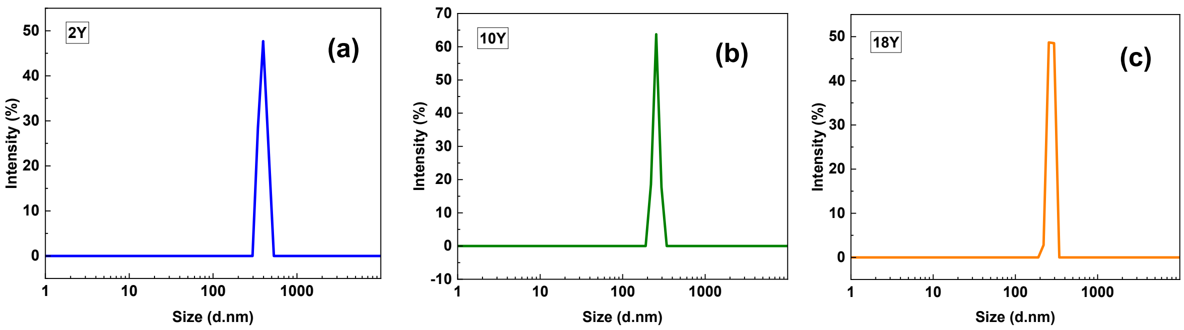

Figure SI4. DLS of (a) 2Y (b) 10Y (c) 18Y

Table of DLS Measurements

Sample	Z-Average (d.nm)	PDI	Peak 1 (Size d.nm)	SD (d.nm) or Error bars	Zeta Potential (mV)
**2Y**	1388	0.863	395.8	± 42.05	-11.4
**10Y**	1579	0.914	255.7	± 22.63	-9.67
**18Y**	1319	0.964	273.6	± 21.84	-8.15
